# Preoperative diagnosis of a gastric extremely well-differentiated adenocarcinoma: A case report

**DOI:** 10.1016/j.ijscr.2020.07.050

**Published:** 2020-07-18

**Authors:** Katsushi Suenaga, Shiro Matsumoto, Alan Kawarai Lefor, Yoshimasa Miura, Yoshinori Hosoya, Daigo Kuboki, Hidenori Haruta, Kentaro Kurashina, Atsushi Kihara, Daisuke Matsubara, Yasunari Sakuma, Joji Kitayama, Naohiro Sata

**Affiliations:** a Departments of Surgery, Jichi Medical University, Tochigi, Japan; b Departments of Gastroenterology, Jichi Medical University, Tochigi, Japan; c Departments of Diagnostic Pathology, Jichi Medical University, Tochigi, Japan

**Keywords:** EWDA of the stomach, Submucosal tumor, Endoscopic submucosal dissection, Preoperative biopsy, Case report

## Abstract

**Introduction:**

Gastric adenocarcinomas with low grade atypia may be difficult to diagnose as gastric cancer by preoperative biopsy. We report an extremely well-differentiated adenocarcinoma (EWDA) of the stomach which appeared like a submucosal tumor diagnosed by preoperative endoscopic submucosal dissection.

**Presentation of case:**

A 70-year-old male was referred with a 3-month history of a submucosal-appearing lesion in the gastric wall found on endoscopy. Biopsies of the lesion were performed and were inconclusive for neoplasia. Endoscopic ultrasonography showed a low echoic tumor growing into the fourth layer of the gastric wall. It was difficult to identify the tumor by repeat biopsy. Endoscopic submucosal dissection of the lesion was performed and revealed adenocarcinoma, and laparoscopic total gastrectomy was performed. Histopathologic evaluation showed that the tumor was stage IIA (T3N0M0). There is no recurrence 12 months after resection.

**Discussion:**

Gastric EWDAs are rare lesions, accounting for 0.6% of all gastric cancers. It is difficult to diagnose gastric EWDA especially if it appears like a submucosal tumor. This lesion was finally diagnosed by endoscopic submucosal dissection.

**Conclusion:**

Endoscopic submucosal dissection may facilitate establishing the preoperative diagnosis of a tumor thought to be a gastric EWDA based on its endoscopic appearance and pathological findings.

## Highlights

• Gastric extremely well-differentiated adenocarcinoma (EWDA) is a rare type of gastric adenocarcinoma.
• It is very difficult to preoperatively diagnose gastric EWDA presenting as an elevated lesion appearing like a submucosal tumor.
• This is the first report of a gastric EWDA appearing like a submucosal tumor diagnosed by preoperative endoscopic submucosal dissection.

## 1 Introduction

Some gastric adenocarcinomas have low-grade nuclear and structure atypia. Gastric extremely well-differentiated adenocarcinoma (EWDA) is defined as a neoplastic lesion consisting of highly differentiated neoplastic epithelium which mimics normal gastric mucosa or intestinal metaplastic mucosa with mild nuclear atypia, but has the ability to invade the gastric wall [[1], [2], [3], [4]]. It also can be classified into gastric and intestinal phenotypes [[1], [2], [3], [4]]. It is located in the middle or upper third of the stomach and appears like a submucosal tumor [1]. It has been reported that they are difficult to diagnose as gastric cancer by preoperative biopsy [5].

A patient presented with a lesion that could not be characterized despite repeated preoperative biopsies including a boring biopsy. Gastric EWDA was suspected based on the endoscopic appearance like a submucosal tumor in the proximal gastric body and the lesion was finally identified after endoscopic submucosal dissection. To the best of our knowledge this is the first report of a gastric EWDA which appeared like a submucosal tumor diagnosed by endoscopic submucosal dissection.

This work is reported in accordance with SCARE criteria [6].

## 2 Presentation of case

**Fig. 1:**
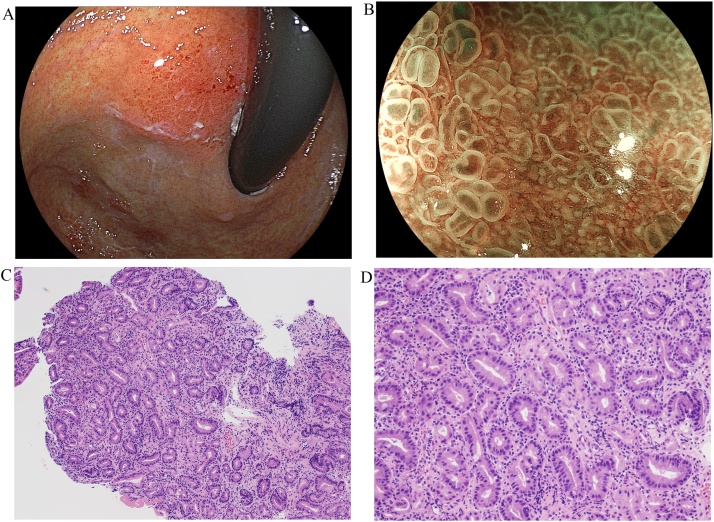
A, B, C, D. Endoscopic examination showed a submucosal appearing lesion in the posterior wall of the proximal gastric body (1A). The lesion had an irregular microsurface pattern and microvascular pattern in the magnifying blue laser imaging (1B). Biopsy revealed atypical glands without architectural atypia and nuclear atypia (H&E, x100 (1C), x200 (1D)).

**Fig. 2:**
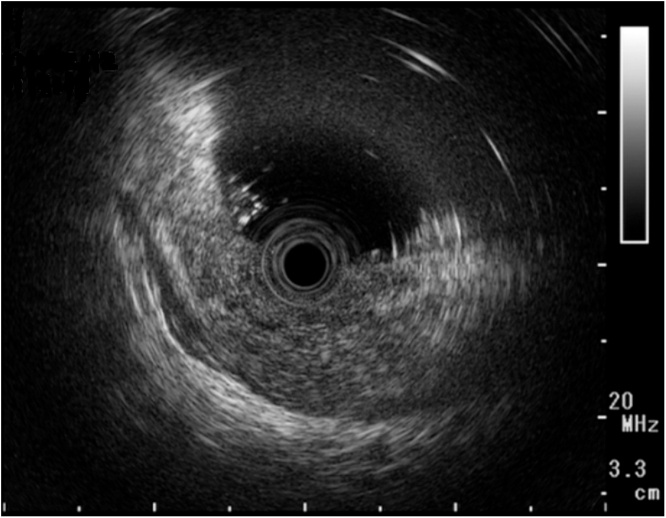
Endoscopic ultrasonography showed a low echoic tumor extending into the fourth layer of the gastric wall.

A 70-year-old male was referred with a 3-month history of a depressed lesion on the posterior wall of the proximal gastric body at a routine endoscopic examination. At the age of 67, he underwent *Helicobacter pylori* eradication therapy which was confirmed negative 3 months later. The first endoscopic examination revealed a lesion with depressions in the center on the surface, but protruding as a whole and appeared like a submucosal tumor, located on the posterior wall of the proximal gastric body (Fig. 1A). It had an irregular microsurface pattern and microvascular pattern using magnifying blue laser imaging (Fig. 1B). A biopsy showed gastric mucosa with intestinal type glands and minimal architectural abnormalities. The glands were comprised of eosinophilic columnar epithelium and a few scattered goblets cells with low-grade nuclear atypia (Fig. 1C, D). Immunohistochemical expression of p53 had a normal pattern. Although the diagnosis of EWDA was considered, reactive intestinal metaplasia was not excluded. Endoscopic ultrasonography showed a low echoic tumor growing into the fourth layer of the gastric wall (Fig. 2). Computed tomography scan did not show the tumor in the gastric wall and there was no evidence of metastases.

**Fig. 3:**
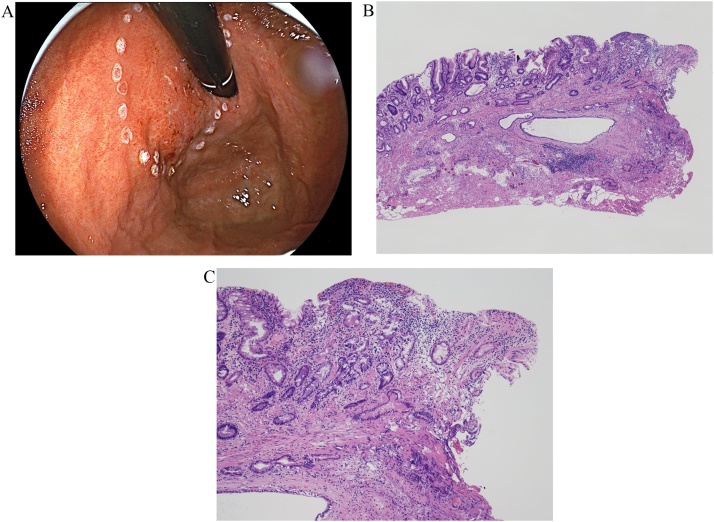
A, B, C. Endoscopic submucosal dissection was of the lesion was performed (3A). The histopathologic examination of the specimen showed acidophilic columnar cancer cells indicating mild to moderate nuclear atypia infiltrated deeper than the submucosal layer with forming mucinous glands clearly. Both horizontal and vertical margins were positive (H&E, x40 (3B), x100 (3C)).

**Fig. 4:**
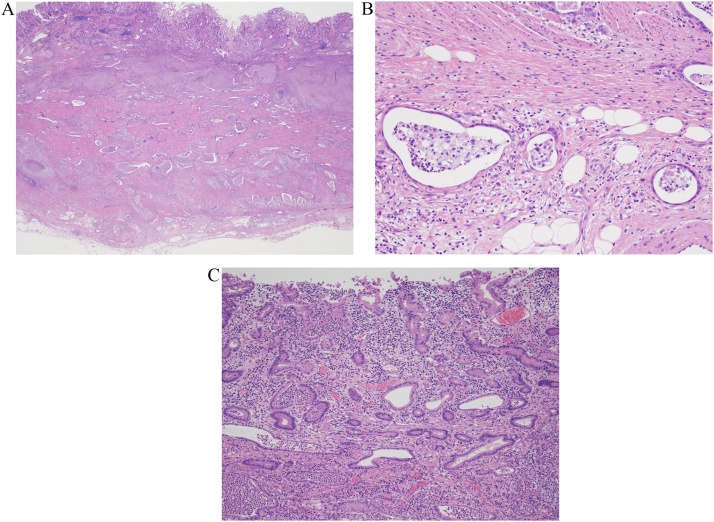
A, B, C. Laparoscopic total gastrectomy was performed. The resected tumor had acidophilic columnar cancer cells indicating mild to moderate nuclear atypia infiltrating the mucosal layer to the subserosa with clear formation of mucinous glands (H&E, x12.5 (4A), x200 (4B), x100 (4C)).

Although we considered that the tumor could be a gastric EWDA because of its endoscopic appearance, it was impossible to conclusively diagnose gastric cancer despite repeated preoperative biopsies including a boring biopsy. We thought that it was important to obtain a sufficient material including the deeper submucosal layer. Therefore, as a partial dissection to obtain an adequate sample of tissue from deeper in the lesion, an endoscopic submucosal dissection to establish a definitive diagnosis was performed (Fig. 3A). Histologically, intestinal-type glands were growing into the submucosa, and the lesion diagnosed definitively as adenocarcinoma. Compared to the intramucosal component, infiltrating glands showed heterogeneous architecture with a thin epithelium and dilated lumen. The tumor had obvious involvement of both the lateral and vertical margins indicating incomplete dissection (Fig.3B, C). The patient subsequently underwent laparoscopic total gastrectomy with lymph node dissection. Pathological examination of the resected stomach revealed that the tumor infiltrated the subserosa with moderate vascular invasion (Fig. 4A–C). Both proximal and distal margins were tumor free. No metastases were found in the lymph nodes. The tumor was finally classified as stage IIA (T3N0M0) according to the 2018 AJCC system. There is no evidence of recurrence 12 months after resection.

## 3 Discussion

Gastric EWDAs are a rare type of gastric adenocarcinoma, which account for just 0.6% of all gastric cancers and for 0.1% of well-differentiated gastric cancers. It is reported that using a combination of at least three of six architectural features, including anastomosing glands, spiky glands, distended glands, discohesive cells, abortive glands, and glandular outgrowth is informative and useful for diagnosing intestinal-type EWDA.

The intestinal immunophenotype has a less aggressive biological behavior which is supported by a low Ki-67 and a lack of p53 or c-erbB2 protein overexpression [1,2].

A study showed that in 31 patients with gastric EWDA, 5 were diagnosed by, and 8 were suspected by, preoperative biopsy. In 7 patients, the lesions were similar to submucosal tumors [7]. Another study showed that in 9 patients reported with gastric EWDA, the tumors were located in the proximal or middle third of the stomach. They were polypoid masses appearing like Borrmann type 1 lesions or submucosal tumors mimicking Bormann type 4. It is difficult to definitively diagnose gastric EWDA by preoperative biopsy, especially in patients with Borrmann type 4 lesions [5].

In some patients, gastric cancer is advanced when the definitive diagnosis is established. In early gastric EWDA, the border between the normal mucosa and the cancer is difficult to distinguish. Therefore, the lateral margin tends to be positive if endoscopic submucosal dissection is performed for treatment and the complete excision rate by endoscopic submucosal dissection is low [8,9].

To date, only 14 patients (including the present patient) with gastric EWDA with an elevated lesion appearing like a submucosal tumor have been reported. The pathological findings, operative procedure, and initial diagnostic procedures for these patients are summarized in Table 1. Patients ranged from 42 to 77 years old and 12/14 were male. The diagnosis was established by preoperative biopsy in 0/14 patients. In most patients, resection was performed without a definitive preoperative diagnosis and diagnoses were made from the surgically excised specimen. Diagnoses were made by frozen section in 2/14 and after laparoscopic partial resection in 1/14. In the present patient, the diagnosis made after endoscopic submucosal dissection of the lesion. Gastric EWDA was suspected based on endoscopic findings. However, in all specimens obtained preoperatively, by endoscopic submucosal dissection and surgical resection, there were no architectural features in the gastric mucosa retrospectively and it was difficult to diagnose by preoperative biopsy. However, endoscopic submucosal dissection of the lesion showed findings of infiltration and the six architectural features in the submucosa, diagnosing gastric EWDA. It is important to assess the findings in the submucosa.

**Table 1:** Previous reports of gastric extremely well-differentiated adenocarcinoma appearing like a submucosal tumor (1989–2018) n = 14.

Patient Number	Reference	Age (years)/Gender	Preoperative biopsy	Depth of invasion	Operative Procedure	First diagnostic procedure
1	Yaosaka et al. [10]	53/M	Indefinite for neoplasia	MP	Distal gastrectomy	Excised specimen
2	Takahashi et al. [11]	69/M	Normal	SS	Distal gastrectomy	Excised specimen
3	Matsunaga et al. [12]	42/M	Indefinite for neoplasia	SM	Distal gastrectomy	Excised specimen
4	Kobayashi et al. [13]	55/M	Suspicious for adenocarcinoma	SS	Total gastrectomy	Excised specimen
5	Adachi et al. [14]	54/F	Normal	SM	Distal gastrectomy	Intraoperative consultation
6	Ono et al. [15]	69/M	Indefinite for neoplasia	SI	Total gastrectomy	Excised specimen
7	Sato et al. [16]	50/M	Normal	SS	Distal gastrectomy	Excised specimen
8	Nobuki et al. [17]	60/M	Normal	SI	Total gastrectomy	Intraoperative consultation
9	Yamamoto et al. [18]	57/M	Not assessed	MP	Total gastrectomy	Excised specimen
10	Ishibashi et al. [7]	57/M	Suspicious for adenocarcinoma	SM	Distal gastrectomy	Excised specimen
11	Okino et al. [19]	66/F	Indefinite for neoplasia	SS	Laparoscopic partial resection	Excised specimen
12	Katada et al. [20]	51/M	Adenoma	MP	Distal gastrectomy	Excised specimen
13	Ohara et al. [8]	64/M	Indefinite for neoplasia	SS	Total gastrectomy	Excised specimen
14	Present patient	77/M	Indefinite for neoplasia	SS	Endoscopic submucosal dissection → Laparoscopic total gastrectomy	Endoscopic submucosal dissection

M, male; F, female; MP, muscularis propria; SS, subserosa; SM, submucosa; SI, invasion of adjacent organ.

## 4 Conclusion

In patients with lesions without definitive pathological findings on biopsy, the diagnosis of a lesion suspected to be a gastric EWDA can be made based on endoscopic findings using endoscopic submucosal dissection to assess the findings in the submucosa. Due to their growth characteristics, formal resection may subsequently be needed.

## Declaration of Competing Interest

The authors declare no conflicts of interest or competing interests.

## Funding

None.

## Ethical approval

Review of this case report was waived by the Jichi Medical University Institutional Review Board.

## Consent

Written and signed consent was given by the patient for this case report.

## Author contribution

Katsushi Suenaga: Conception of Study, Acquisition and analysis of data, drafting and critical revisions of the article, approval of final version.

Shiro Matsumoto: Acquisition of data, critical revisions of the article, approval of final version.

Alan Kawarai Lefor: Acquisition of data, critical revisions of the article, approval of final version.

Yoshimasa Miura: Acquisition of data, critical revisions of the article, approval of final version.

Yoshinori Hosoya: Acquisition of data, critical revisions of the article, approval of final version.

Daigo Kuboki: Acquisition of data, critical revisions of the article, approval of final version.

Hidenori Haruta: Acquisition of data, critical revisions of the article, approval of final version.

Kentaro Kurashina: Acquisition of data, critical revisions of the article, approval of final version.

Atsushi Kihara: Acquisition of data, critical revisions of the article, approval of final version.

Daisuke Matsubara: Acquisition of data, critical revisions of the article, approval of final version.

Yasunari Sakuma: Acquisition of data, critical revisions of the article, approval of final version.

Joji Kitayama: Acquisition of data, critical revisions of the article, approval of final version.

Naohiro Sata: Acquisition of data, critical revisions of the article, approval of final version.

## Registration of research studies

Not Applicable.

## Guarantor

Katsushi Suenaga MD.

## Provenance and peer review

Not commissioned, externally peer-reviewed.
